# Protective effects of *Fraxinus xanthoxyloides* (Wall.) leaves against CCl_4_ induced hepatic toxicity in rat

**DOI:** 10.1186/s12906-016-1398-0

**Published:** 2016-10-24

**Authors:** Tahira Younis, Muhammad Rashid Khan, Moniba Sajid

**Affiliations:** Department of Biochemistry, Faculty of Biological Sciences, Quaid-i-Azam University, Islamabad, 45320 Pakistan

**Keywords:** *Fraxinus xanthoxyloides*, Antioxidant, Phenolics, Liver, CCl_4_, Lipid peroxidation

## Abstract

**Background:**

Leaves and root bark of *Fraxinus xanthoxyloides* Wall. (Oleaceae) are used locally for the treatment of jaundice, malaria and pneumonia. Decoction of stem, twigs and bark is used in pain, internal injuries, rheumatism and in bone fracture. In this investigation we have evaluated the methanol extract of leaves for its hepatoprotective potential against CCl_4_ induced hepatic injuries in rat.

**Methods:**

Powder of *F. xanthoxyloides* leaves was extracted with methanol (FXM) and subjected for the determination of polyphenolics through HPLC-DAD analysis. Sprague–Dawley (*Rattus novergicus*) male rats were divided into eight groups (six rats in each). Group I: non-treated control; Group II: vehicle treated (DMSO plus olive oil) while Group III- VI treated with 1 ml/kg body weight (bw) of CCl_4_ (30 % in olive oil) for 30 days (15 dosages) to induce the hepatic injuries. Group IV: treated with silymarin (100 mg/kg bw); Group V and VI with FXM (200, 400 mg/kg bw) on alternate days with CCl_4_ treatment. Group VII and VIII was administered with FXM (200, 400 mg/kg bw) alone (15 dosages). After 30 days the serum was evaluated for liver function enzymes and biochemical markers, liver samples for antioxidant enzymes, biochemical markers, comet assay and for histopathology.

**Results:**

HPLC-DAD analysis of FXM revealed the existence of rutin and caffeic acid. In CCl_4_ treated rats the level of alanine transaminase (ALT), aspartate transaminase (AST), total bilirubin was significantly increased while the albumin concentration in serum was decreased as compared to control group. The level of hepatic antioxidant enzymes, catalase (CAT), peroxidase (POD), superoxide dismutase (SOD), glutathione-S-transferase (GST) and glutathione reductase (GSR) was significantly decreased against the control group. Further, significant decrease in GSH while increase in lipid peroxides (TBARS), H_2_O_2_, DNA damages and comet length was induced with CCl_4_ in hepatic tissues of rat. In contrast, co-administration of FXM and silymarin restored the biochemical and histopathological status of the liver.

**Conclusion:**

Results of present investigation revealed that *F. xanthoxyloides* leaves possibly protect the liver against CCl_4_ induced injuries like silymarin by its antioxidant constituents.

## Background

Plants are used traditionally for the treatment and prevention of several human diseases and are thought to play their undeniable role in health care system. In living systems free radicals such as superoxide anion (O2^•-^), hydroxyl radicals (OH•), singlet oxygen (^1^O_2_) and other reactive oxygen species like hydrogen peroxide (H_2_O_2_) are known to play their deleterious role and have damaging effects on different cellular organelles. These are supposed to be the main cause of pathogenesis of different human diseases like cancer, atherosclerosis, diabetes mellitus, arthritis, Alzheimer’s disease and Parkinsonism. High concentrations of ROS within the cell leads to destruction and disruption of lipids of the membranous system, cellular proteins and causes DNA damage thus causes several diseases [1, 2].

Antioxidants are the compounds capable of preventing or reducing harmful effects of free radicals. The exogenous antioxidants mainly consist of synthetic and natural antioxidants. Treatment of different diseases using exogenous antioxidants is of prime importance but due to side effects of synthetic antioxidants their use is limited. There have been increasing safety concerns over synthetic antioxidants. For example, butylated hydroxyanisole (BHA) and butylated hydroxytoluene (BHT), the two well-known synthetic antioxidants, have been restricted for their DNA damaging and other toxic effects [3]. Moreover it has been also determined that natural antioxidants of vegetables and fruits prevent different diseases [4].

Hepatic diseases are the most common of all the pathologies worldwide and constitute up to 83 % of all the cases. Among the hepatic pathologies toxicity is the most common risk factor which is usually contributed by food additives, alcohol, toxic industrial chemicals, air and water pollutants. Carbon tetrachloride (CCl_4_) is a toxin used in animal models that mimics the oxidative stress led injuries in various organs [5, 6]. It is metabolized by cytochrome P-450, and produces trichloromethyl (CCl_3_) radical and chlorine. Trichloromethyl radical has the capacity to abstract double allylic hydrogen atoms from polyenoic fatty acids of cellular membranes. This reaction led to the generation of chloroform and highly reactive secondary lipid radicals that react with molecular oxygen to form lipid peroxy radicals. Alternatively the trichloromethyl radical directly reacts with the molecular oxygen to form the peroxytrichloromethyl radical (CCl_3_O_2_) that is even more injurious to that of trichloromethyl radical in the production of lipid peroxy radicals. These lipid peroxydized fatty acids eventually decomposed in to stable carbonyls such as malondialdehyde pentane and ethane [7]. On the other hand trichloromethyl radical can bind with macromolecules and causes massive damages to proteins, DNA and lipids. So CCl_4_ elicits the generation of unsaturated fatty acids, lipoperoxides and free radicals that contribute to the pathological consequences [8].

Treatment of oxidative stress induced pathologies with natural antioxidants is of great concern these days. Daily intake of green leafy vegetables, fresh fruits and tea (*Camellia sinensis*) as a source of natural antioxidants have been found useful for prevention of cardiovascular and neurodegenerative diseases [6, 9, 10]. Among phytochemicals flavonoids are the most prominent for their antioxidant action. Quenching of free radicals is a property of flavonoids imparted by their redox property as a result they act as reducing agents, singlet oxygen scavengers, hydrogen donors and reductants of ferryl hemoglobin [11]. Plants possessing flavonoids exhibit strong anti-inflammatory, antiviral, antioxidant, anti-allergenic, anti-fungal, anti-bacterial, anticancer, cytotoxic and hepatoprotective activities thus, generated curiosity about flavonoid containing plants [12–14].

*Fraxinus xanthoxyloides* (Wall. ex G.Don) DC. (Family Oleaceae) is found in Northern areas of Pakistan, Afghanistan, India, Morocco and in Algeria. It is commonly known as Afghan ash. Local practitioners in northern areas of Pakistan use its leaves and root bark for the treatment of jaundice, malaria and pneumonia [15]. Stem bark of *F. xanthoxyloides* is used in the form of decoction by the local communities to reduce pain during labor [16], expulsion of pre-mature infant after death [17] and also used in traumas [18]. Decoction of stem/twigs is also used in wounds and bone fractures in cattle [19]. Its wood is used by the local people in bone fracture [20]. Its leaves are also used as fodder [15]. Use of the extract of *F. excelsior* seeds in diabetic volunteers had significantly reduced the postprandial rise in glycemia while enhanced the insulin secretion [21]. Treatment of obese mice with extracts of *F. excelsior* seeds limited the gain in weight and hyperglycemia [22]. Further, seed extract of *F. excelsior* protected the micronuclei in irradiated human lymphocytes and did not induce changes in hematological and biochemical parameters after 90 days of its use in human [23]. Administration of 10 mg/kg of methanol extract of the aerial parts of *F. micrantha* produced significant anti-inflammatory effects against carrageenan-induced acute inflammation in mice [24]. Xanthoxyloidin, a new biscoumarin together with esculetin, 5,7- dihydroxycoumarin and 6,8-dihydroxy-7-methoxycoumarin were isolated from the methanol extract of the whole plant of *F. xanthoxyloides* [25]. Intraperitoneal administration of stem bark extract of *F. ornus* displayed anti-inflammatory activity in both zymosan- and carrageenan-induced paw edema in mice [26]. Significant anti-nociceptive and anti-inflammatory activities of the methanol extract at 200 and 400 mg/kg doses of *Fraxinus floribunda* leaves have been reported [27]. The ethanol extract of the aerial parts of *F. rhynchophylla* attenuated the liver fibrosis induced with CCl_4_ in rat probably through free radical scavenging abilities [28]. Streptozotocin and paracetamol induced diabetes and the liver injuries have been attenuated by the treatment of leaves and stem bark extract of *F. angustifolia* in mice. Treatment of mice with both extracts had diminished the lipid peroxidation and increase in biochemical markers of serum in streptozotocin and paracetamol induced damages in mice. The results suggest that hepato-protective and antidiabetic proficiencies might have been established by the presence of antioxidant constituents in the plant [29]. *In vitro* antioxidant activities of the various aqueous extracts from the bark of *F. floribunda* have also been reported [30]. Antioxidant capabilities of the extracts from leaves and stem bark of *F. angustifolia* have also been appraised through *in vitro* studies [31]. Moulaoui et al. [32] also investigated the wound healing potential of *F. angustifolia*. Inner stem bark of *F. micrantha* has been used by the local communities of Dharchula, India for liver enlargement, jaundice and other liver disorders [33]. Liver diseases such as jaundice, enlargement, fibrosis are usually induced by oxidative stress and inflammatory processes [1, 2]. Based on the studies reported earlier for the use of *Fraxinus* species in liver and anti-inflammatory disorders it was speculated that the traditional use of *F. xanthoxyloides* in jaundice by the local communities of Pakistan might be attributed through the antioxidant abilities of the phyto-constituents. In this perspective that natural antioxidant play a role in hepato-protection, the present study was undertaken to evaluate the methanol extract of *F. xanthoxyloides* leaves for its hepato-protective properties against the CCl_4_ induced hepatic toxicity in rat. For the purpose biomarkers of serum, liver homogenate and histopathology was investigated. Further, HPLC-DAD analysis of the FXM was carried out to reveal the presence of flavonoids.

## Methods

### Plant material

The leaves of *F. xanthoxyloides* were collected in October, 2013 from the campus of Quaid-i-Azam University Islamabad, Pakistan. The plant was recognized by its local name and then validated by Dr. Rizwana Aleem Qureshi, Department of Plant Sciences, Quaid-i-Azam University Islamabad. Specimen was (45679) submitted to National Herbarium, Quaid-i-Azam University, Islamabad, Pakistan.

### Preparation of crude extract and fractions

After collection, plant samples were shade dried and powdered by using grinder. Powder (1 kg dry weight) was soaked in 4 l of 95 % crude methanol for 72 h and repeated the above procedure twice. For the purpose of filtration, Whatman No. 1 filter was used and methanol was evaporated on a rotary evaporator at 40 °C under reduced pressure. Extract (FXM) was stored at 4 °C for further investigation.

### High performance liquid chromatography (HPLC) analysis

Presence of polyphenolic components of the FXM was detected by the HPLC-DAD analysis. The apparatus used was of Agilent Germany and analytical column was of Sorbex RXC8 (Agilent USA) with 5 μm particle size and 25 ml capacity. Mobile phase consisted of eluent A, (acetonitrile-methanol–water-acetic acid /5: 10: 85: 1) and eluent B (acetonitrile-methanol-acetic acid/40: 60: 1). The gradient (A: B) utilized was the following: 0–20 min (0 to 50 % B), 20–25 min (50 to 100 % B), and then isocratic 100 % B (25–40 min) at flow rate of 1 ml/min. The injection volume of the sample was 20 μl. Before injection the samples were filtered through 0.45 μm membrane filter. Among the standards rutin and gallic acid were analyzed at 257 nm, catechin at 279 nm, caffeic acid at 325 nm and quercetin, myricetin, kaempferol were analyzed at 368 nm. Every time column was reconditioned for 10 min before the next analysis. All samples were assayed in triplicate. Quantification was carried out by the integration of the peak using the external standard method. All chromatographic operations were carried out at an ambient temperature.

## Animal studies

### CCl_4_ induced hepatic injuries

Protective effects of FXM on the CCl_4_ induced liver injuries were evaluated on healthy male Sprague Dawley (150–200 g) (*Rattus novergicus*) rats with mean age of 2 months. Experimental animals (48) were randomly divided into eight groups having six rats in each. The animals were placed in conventional steel cages at room temperature with 12 h light and dark cycle at the Primate Facility of Quaid-i-Azam University, Islamabad. The experimental protocol (Bch#271) for the use of animal was approved by the ethical board of Quaid-i-Azam University, Islamabad. Animals were fed on rodent chow and tap water *ad libitum*. Protocol of Shyu et al. [34] was followed to carry out this experiment. Animals of Group I were remained untreated (control), Group II as vehicle control (1 ml/kg bw; 10 % DMSO in olive oil, orally). Rats of Group III - VI were administered CCl_4_ (1 ml/kg bw; 30 % CCl_4_ in olive oil) orally on alternative days for 30 days (15 dosages). Group IV was also treated with silymarin (100 mg/kg bw) while Group V and VI with FXM (200, 400 mg/kg bw) on alternate days with CCl_4_ treatment (15 dosages, orally). FXM alone (200, 400 mg/kg bw) was administered to Group VII and Group VIII for 30 days (15 dosages). The dosages of FXM to rats were based on the studies reported earlier [35]. All the treatments were carried out in the morning. Before dissection, all rats were kept on normal feed without any treatment for at least 24 h. Rats were euthanized after ether anesthesia. By using 23 G1 syringes, cardiac puncture was done and blood samples were collected in falcon tubes. Falcon tubes were centrifuged at 500 × g for 15 min at 4 °C and sera were collected for biochemical analysis. Liver was dissected out, rinsed with ice cold saline to remove debris. A part of liver after drying in liquid nitrogen was stored at −70 °C for tissue homogenate tests. Small part of organs was stored in 10 % phosphate buffered formalin for comet assay and histopathological studies.

### Serum analysis

For the analysis of serum samples of rats, the diagnostics kits of AMP (Krenngasse 12, 8010 Graz, Australia) were used to estimate AST, ALT, total bilirubin, albumin and globulin level in serum samples.

### Antioxidant enzymes assessment

An amount of 100 mg of hepatic tissue of each animal was homogenized in 1 ml of potassium phosphate buffer (100 mM) containing EDTA (1 mM) at pH 7.4. The centrifugation of homogenate was done at 12,000 × g at 4 °C for 30 min to obtain the supernatant for following antioxidant enzyme assays.

#### Catalase (CAT) activity

For the CAT activity determination, the protocol of Chance and Maehly [36] was followed. The CAT reaction solution consisted of 625 μl of 50 mM of potassium phosphate buffer (pH 5), 100 μl of 5.9 mM H_2_O_2_ and 35 μl of the supernatant. After one minute, changes in absorbance of the reaction mixture at 240 nm were recorded. One unit of catalase activity was stated as an absorbance change of 0.01 as units/min.

#### Peroxidase (POD) activity

Activity of POD was assayed by Chance and Maehly [36] protocol with slight modifications. POD reaction solution contained 75 μl of 40 mM hydrogen peroxide, 25 μl of 20 mM guaiacol, 625 μl of 50 mM potassium phosphate buffer (pH 5.0) and 25 μl of supernatant. After an interval of one minute, change in absorbance was determined at 470 nm. One unit POD activity is equivalent to change in absorbance of 0.01 as units/min.

#### Superoxide dismutase (SOD) activity

Activity level of SOD was estimated by the protocol of Kakkar et al. [37]. By using phenazine methosulphate and sodium pyrophosphate buffer SOD activity was assessed. Centrifugation of tissue homogenate was done at 1500 × g for 10 min and then at 10,000 × g for 15 min. Supernatant was collected and 150 μl of it was added to the aliquot containing 600 μl of 0.052 mM sodium pyrophosphate buffer (pH 7.0) and 50 μl of 186 mM of phenazine methosulphate. In the end to initiate enzymatic reaction, 100 μl of 780 μM NADH was added. After 1 min, glacial acetic acid (500 μl) was added to stop the reaction. At 560 nm absorbance was determined to enumerate the color intensity. Results were evaluated in units/mg protein.

#### Glutathione-S-transferase (GST) activity

Protocol of Habig et al. [38] was followed for the estimation of GST activity. The assay was based on formation of 1-chloro-2,4-dinitrobenzene (CDNB) conjugate. A volume of 150 μl of tissue supernatant was added to 720 μl of sodium phosphate buffer together with 100 μl of reduced glutathione (1 mM) and 12.5 μl of CDNB (1 mM). By spectrophotometer, absorbance was recorded at 340 nm. Through molar coefficient of 9.61 × 10^3^/M/cm, GST activity was determined as amount of CDNB conjugate formed per minute per mg protein.

#### Glutathione reductase (GSR) activity

The glutathione reductase activity in the hepatic tissues was assessed by the method of Carlberg and Mannervik [39]. GSR activity was based on the conversion of oxidized glutathione (GSSG) into reduced glutathione (GSH) at the expense of NADPH. Briefly, 50 μl of liver supernatant was added to a reaction mixture consisting of 25 μl of 1 mM oxidized glutathione (GSSG), 50 μl of 0.5 mM EDTA and 825 μl of 0.1 M sodium phosphate buffer (pH 7.6). Then an aliquot of 50 μl of 0.1 mM NADPH was added to the reaction mixture to initiate the process and decline in absorbance was recorded at 340 nm at 25 °C for 20 min. Using molar extinction coefficient of 6.22 × 10^3^/M/cm, GSR activity was assessed as amount of NADPH oxidized/min/mg protein.

### Estimation of biochemical parameters

#### Reduced glutathione (GSH) estimation

Quantity of GSH in liver tissues was assessed following the protocol of Jollow et al. [40]. Precipitation of tissue homogenate (500 μl) was carried out by the addition of (500 μl) 4 % sulfosalicylic acid. After 1 h of incubation at 4 °C the reaction mixture was centrifuged for 20 min at 1200 × g. An aliquot of 33 μl of the supernatant was added to 900 μl of 0.1 M potassium phosphate buffer (pH 7.4) and 66 μl of 100 mM of 5,5′-dithio-bis(2-nitrobenzoic acid (DTNB). Reaction of GSH with DTNB produced a yellow colored derivative 5′-thio-2-nitrobenzoic acid (TNB). The absorbance of the reaction mixture was recorded at 412 nm. The GSH activity was expressed as μmole GSH/g tissue.

#### Lipid peroxidation assay (TBARS)

Protocol of Iqbal et al. [41] was adopted with slight modifications for the assessment of lipid peroxidation. The reaction mixture consisted of 290 μl of 0.1 M phosphate buffer (pH 7.4), 10 μl of 100 mM ferric chloride, 100 μl of 100 mM ascorbic acid, and 100 μl of homogenized sample. After 1 h incubation of the mixture at 37 °C in shaking water bath, 500 μl of trichloroacetic acid (10 %) was added to inhibit the reaction. Then 500 μl of 0.67 % thiobarbituric acid was added and the reaction tubes were placed in water bath for 20 min. After that the tubes were placed in crushed ice bath for 5 min and centrifugation was done at 2500 × g for 12–15 min. Absorbance of the supernatant was recorded at 535 nm. By using molar extinction coefficient of 1.56 × 10^5^ /M/cm, results were calculated as nmole of TBARS formed per min per mg tissue at 37 °C.

#### Protein assessment

Procedure of Lowry et al. [42] was followed in order to find the total soluble proteins within the tissues. For this purpose, 100 mg of organ was weighed and homogenization was done in potassium phosphate buffer. Homogenized mixture was centrifuged at 4 °C at 10,000 × g for 15–20 min to obtain the supernatant. Alkaline solution 1 ml was added in 0.1 ml of supernatant and mixed vigilantly with the help of vortex machine. Then the incubation was done for 30 min. Afterwards the change in absorbance was calculated at 595 nm. Bovine serum albumin (BSA) curve was used to find out the concentration of serum proteins in the sample.

#### Hydrogen peroxide assay

Estimation of hydrogen peroxide was done by following Pick and Keisari [43] protocol. The H_2_O_2_ horseradish peroxidase enzyme brought about the oxidation of phenol red. In the reaction mixture, 500 μl of 0.05 M phosphate buffer (pH 7),100 μl of homogenate was added along with 100 μl of 0.28 nmole phenol red solution, 250 μl of 5.5 nmole dextrose and horse radish peroxidase (8.5 units) was added. Incubation was done at room temperature for 60 min. A volume of 100 μl of NaOH (10 N) was added to stop the reaction. Then mixture tubes were centrifuged for 5–10 min at 800 × g. By spectrophotometer the absorbance of the collected supernatant was measured against reagent as a blank at 610 nm. Production of H_2_O_2_ was measured as nmole H_2_O_2_/min/mg tissue on the basis of standard curve of H_2_O_2_ oxidized phenol red.

### DNA injuries

#### Comet assay

We have adopted the protocol of Dhawan et al. [44] to monitor the DNA damages through comet assay. Sterilized slides were dipped in hot normal melting agarose (1 %) solution and allowed to solidify at room temperature. A small piece of liver tissue was placed in 1 ml of cold lysing solution and minced in to small pieces and mixed with 75 μl of low melting agarose solution. This mixture was coated on the already coated slides and a cover slip was gently placed over it. The slide was placed on ice packs for about 8–10 min. Cover slip was removed and again low melting point agarose was added and placed on ice packs for solidification. After three coating with low melting point agarose slide was again placed in the lysing solution for about 10 min and placed in refrigerator for 2 h. After electrophoresis slide was stained with 1 % ethidium bromide and visualized under fluorescent microscope. CASP 1.2.3.b image analysis software was used to evaluate the extent of DNA damage. In each sample 50–100 cells were analyzed for comet length, head length, tail length, tail moment and DNA content in head of renal cell’s nuclei.

#### DNA fragmentation assay

Protocol of Wu et al. [45] was adopted for the estimation of DNA injuries in hepatic tissues of rat. A volume of 0.1 ml of the hepatic tissues homogenate in Tris triton EDTA (TTE) labelled B was centrifuged at 200 × g at 48 °C for 10 min. The supernatant collected was labelled S and again centrifuged at 20,000 × g for 10 min at 48 °C. The intact chromatin obtained was labeled C. After addition of 1.0 ml of 25 % TCA to all tubes; B, S and C were incubated overnight at 48 °C. After centrifugation at 18,000 × g at 48 °C the precipitated DNA was recovered. To each tube 160 ml of 5 % TCA was added and heated for 15 min at 90 °C followed by the addition of 320 ml of freshly prepared DPA solution. Each tube was vortexed vigorously and incubated for 4 h at 37 °C. Optical density of the reaction assay was recorded at 600 nm. The results were presented as %fragmented DNA by using the formula:

$$ \mathrm{D}\mathrm{N}\mathrm{A}\;\mathrm{fragmentation}\;\left(\%\right)=\left[\frac{\mathrm{C}\times 100}{\mathrm{C}+\mathrm{B}}\right] $$

### Histopathological examination

For histopathological examination, a fixative containing absolute alcohol (85 ml), glacial acetic acid (5 ml) and 40 % formaldehyde (10 ml) was used to fix hepatic tissues. For slides preparation, thin sections of fresh tissues of liver about 3–4 μm were used. The hematoxylin-eosin stain was used for staining purpose and for histopathological study a light microscope (DIALUX 20 EB) at magnification of 40X was used.

### Statistical analysis

The values were expressed as mean ± standard deviation. For *in vivo* studies, the consequences of different treatments given to animals were evaluated by one way analysis of variance which was carried by means of computer software GraphPad prism 4.0. Multiple comparisons among various treatments were made by Tukeys’ HSD method at P-value ≤ 0.01.

## Results

### HPLC-DAD analysis

**Fig. 1:**
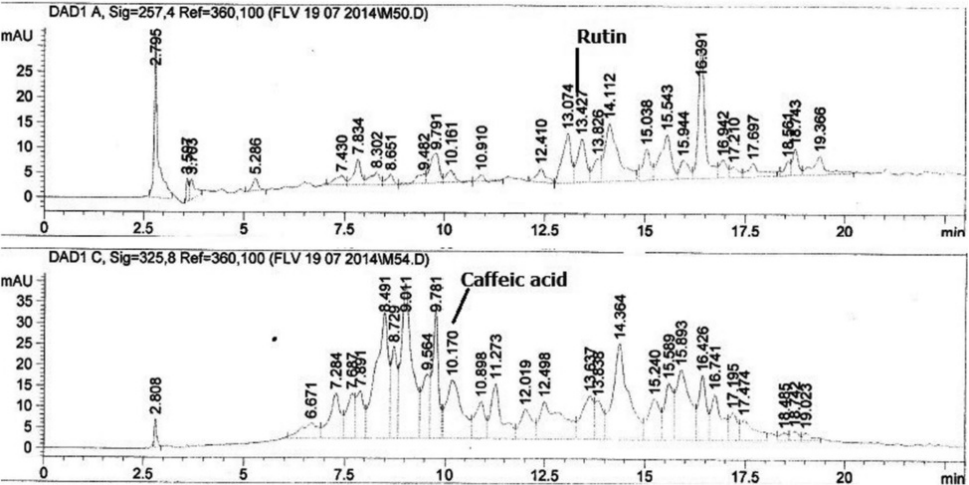
HPLC-DAD profile of the crude methanol extract of *F. xanthoxyloides* (FXM)

Qualitative analysis of the crude methanol extract of *F. xanthoxyloides* leaves was carried out by using HPLC-DAD and their chromatographic profile was compared with the retention times and absorption spectrum of reference standards (rutin, kaempferol, myricetin, gallic acid, catechins, caffeic acid and quercetin). From the HPLC profile it was observed that FXM contains rutin 26.13 ± 1.2 μg/mg and caffeic acid 29.22 ± 2.8 μg/mg of extract (Fig. 1).

### Effect of FXM on serum markers

The results obtained for the treatment of CCl_4_ and with co-administration of FXM on the serum biochemical markers are shown in Table 1. Treatment of CCl_4_ significantly (*P* < 0.001) increased the level of liver function enzymes; AST and ALT as compared to the control group. Multiple comparison among the treatments indicated that the increase in the level of liver function enzymes with CCl_4_ was significantly decreased with the co-administration of silymarin as well as with the both doses of FXM. The activity level of AST and ALT recorded with co-administration of silymarin and the higher dose of FXM (400 mg/kg) was statistically similar to each other. However, administration of FXM alone at both the doses did not induce any significant (*P* > 0.05) change in the serum level of AST and ALT as compared with the control group.

**Table 1:** Effect of FXM on biochemical parameters of serum in rat

Treatment	AST (U/l)	ALT (U/l)	Bilirubin (mg/dl)	Albumin (mg/dl)	Globulin (mg/dl)
Control	86.66 ± 5.35^d^	32.66 ± 4.17 ^d^	0.165 ± 0.024^c^	4.45 ± 0.27^a^	1.55 ± 0.18^c^
DMSO + Olive oil	89.50 ± 6.05 ^d^	33.16 ± 3.65 ^d^	0.160 ± 0.025^c^	4.60 ± 0.17^a^	1.52 ± 0.18^bc^
CCl_4_	319.83 ± 20.22^†a^	204.67 ± 20.91^†a^	0.663 ± 0.132^†a^	2.33 ± 0.31^†b^	2.27 ± 0.10^†a^
CCl_4_ + silymarin (100 mg/kg)	122.00 ± 21.07^≠c^	63.50 ± 5.28^†c^	0.256 ± 0.043^*bc^	3.93 ± 0.21^a^	1.81 ± 0.26^bc^
CCl_4_ + FXM (200 mg/kg)	212.67 ± 29.04^†b^	105.50 ± 6.53^†b^	0.325 ± 0.030^†b^	4.00 ± 0.50^a^	2.13 ± 0.09^†ab^
CCl_4_ + FXM (400 mg/kg)	116.83 ± 12.25^*cd^	61.33 ± 6.21^†c^	0.161 ± 0.030^c^	4.23 ± 0.47^a^	2.09 ± 0.23^†ab^
FXM (200 mg/kg)	84.83 ± 5.07 ^d^	36.66 ± 3.66 ^d^	0.156 ± 0.021^c^	4.35 ± 0.18^a^	1.51 ± 0.14^c^
FXM (400 mg/kg)	86.66 ± 4.88 ^d^	33.16 ± 3.06 ^d^	0.161 ± 0.023^c^	4.43 ± 0.32^a^	1.60 ± 0.14^c^

*FXM F. xanthoxyloides* methanol extract. Data values shown represent mean ± SD (*n* = 6). One way analysis of variance was followed by Dunnet comparison of various treatments with control at: ^*^, *P* < 0.05; ^#^, *P* < 0.01; ^ǂ^, *P* < 0.001. Multiple comparisons among treatments were determined by Tukeys’ HSD test. Superscript alphabets indicate significance among treatments (*P* < 0.01) on not sharing common letter

In this experiment serum level of total bilirubin was significantly (*P* < 0.001) increased with CCl_4_ treatment as compared to the control group. The toxicity induced with CCl_4_ was ameliorated by the co-administration of silymarin and also at both doses of FXM. On the other hand serum level of total bilirubin was decreased drastically (*P* < 0.01) at higher dose of FXM (400 mg/kg) and did not differ from the control group. Serum level of the total bilirubin of the groups treated with FXM alone (200, 400 mg/kg) remained statistically (*P* > 0.05) similar with the control group.

In animals treated with CCl_4_ the level of albumin was decreased (*P* < 0.001) whereas the level of globulin was increased (*P* < 0.001) as compared to the control group. Co-administration of silymarin and the two doses of FXM attenuated the CCl_4_ intoxication and the level of the albumin and the globulin in serum was restored towards the control level. Multiple comparisons of the treatments exhibited that the level of albumin and globulin in serum of silymarin and FXM (both doses) treated groups was statistically (*P* > 0.01) similar among each other and also with control group. Co-administration of silymarin produced more marked ameliorating effects on the toxicity induced with CCl_4_ and the level of globulin in serum did not differ from the control group. However, the level of globulin obtained with co-administration of FXM was significantly lower to that of the control group. The results indicated that the treatment of FXM alone did not induce significant (*P* > 0.05) changes in the albumin and the globulin as compared to the control group.

### Effect of FXM on antioxidant enzymes of liver

Table 2 indicates significant (*P* < 0.001) decrease in the activity level of antioxidant enzymes of liver; CAT, POD, SOD, GST and GSR as compared to the control group. Prophylactic treatment of FXM and silymarin reduced the toxicity of CCl_4_ and the activity level of hepatic antioxidant enzymes was restored towards the control group. Multiple comparisons of various treatments indicated that low dose of FXM (200 mg/kg) was not much effective in ameliorating the oxidative stress induced with CCl_4_ so that the activity level of all the hepatic antioxidant enzymes evaluated in this experiment showed lower activity to that of the control group. The activity level of antioxidant enzymes was significantly (*P* < 0.01) restored by the co-administration of the higher dose of FXM (400 mg/kg) and with silymarin. However, treatment of FXM alone at both the doses; 200 and 400 mg/kg alone did not alter the level of these parameters as compared to the control.

**Table 2:** Effect of FXM on antioxidant enzymes of liver in rat

Treatment	CAT (U/min)	POD (U/min)	SOD (U/mg protein)	GST (mM/min/mg protein)	GSR (mM//min/mg protein)
Control	3.88 ± 0.53^ab^	9.55 ± 0.99^ab^	3.00 ± 0.31^a^	160.81 ± 8.53^a^	224.62 ± 9.02^a^
DMSO + Olive oil	4.19 ± 0.27^a^	10.65 ± 1.44^ab^	3.07 ± 0.27^a^	158.56 ± 11.67^a^	220.11 ± 8.01^a^
CCl_4_	2.33 ± 0.18^†c^	5.31 ± 0.63^†c^	1.30 ± 0.23^†c^	100.11 ± 4.99^†c^	130.52 ± 9.77^†d^
CCl_4_ + silymarin (100 mg/kg)	3.88 ± 0.28^ab^	9.31 ± 0.67^ab^	2.93 ± 0.23^ab^	151.02 ± 9.49^ab^	180.88 ± 9.17^†c^
CCl_4_ + FXM (200 mg/kg)	2.64 ± 0.15^†c^	7.45 ± 0.39^†b^	1.58 ± 0.11^†c^	133.85 ± 8.34^†b^	184.83 ± 8.90^†c^
CCl_4_ + FXM (400 mg/kg)	3.37 ± 0.24^*b^	9.01 ± 0.55^ab^	2.46 ± 0.19^≠b^	150.70 ± 8.98^ab^	198.03 ± 10.64^†bc^
FXM (200 mg/kg)	3.79 ± 0.23^ab^	9.44 ± 0.87^ab^	2.99 ± 0.18^a^	159.57 ± 9.57^a^	221.17 ± 7.15^a^
FXM (400 mg/kg)	3.91 ± 0.45^ab^	9.59 ± 0.75^ab^	2.94 ± 0.20^ab^	159.66 ± 9.63^a^	216.03 ± 8.42^ab^

*FXM F. xanthoxyloides* methanol extract. Data values shown represent mean ± SD (*n* = 6). One way analysis of variance was followed by Dunnet comparison of various treatments with control at: ^*^, *P* < 0.05; ^#^, *P* < 0.01; ^ǂ^, *P* < 0.001. Multiple comparisons among treatments were determined by Tukeys’ HSD test. Superscript alphabets indicate significance among treatments (*P* < 0.01) on not sharing common letter

### Effect of FXM on biochemical markers of liver

The toxic effects of CCl_4_ and the protective potential of FXM on the biochemical parameters of liver are presented in Table 3. Treatment of CCl_4_ increased the concentration of lipid peroxides (TBARS), H_2_O_2_ and DNA injuries while decreased (*P* < 0.001) the protein and GSH content in the liver tissues as compared to the control group. Co-administration of FXM at its both level of dosages decreased the content of TBARS, H_2_O_2_ and DNA injuries as compared to the CCl_4_ group. Protein and GSH concentration in the hepatic tissues was also increased by the co-treatment of FXM (at both doses) and with silymarin. Multiple comparison among different treatments indicated that restoration potential of FXM (400 mg/kg) and silymarin on TBARS, H_2_O_2_ and the GSH content towards the control was statistically similar to each other. Administration of FXM (both doses) alone did not induce alteration (*P* > 0.05) in the concentration of the above biochemical markers of liver as compared to the control group.

**Table 3:** Effect of FXM on biochemical parameters of liver in rat

Treatment	Protein (μg/mg tissue)	GSH (μmole/g tissue)	TBARS (nmole/min/mg tissue)	H_2_O_2_ (nmole/min/mg tissue)	DNA injury (%)
Control	2.98 ± 0.13^a^	21.05 ± 2.18^abc^	2.96 ± 0.28^d^	2.49 ± 0.09^c^	2.51 ± 0.15^d^
DMSO + Olive oil	2.98 ± 0.09^a^	21.35 ± 2.16^ab^	3.24 ± 0.36^d^	2.60 ± 0.19^bc^	2.58 ± 0.15^d^
CCl_4_	1.05 ± 0.14^†c^	11.38 ± 0.93^†e^	6.19 ± 0.65^†a^	3.99 ± 0.33^†a^	6.22 ± 0.63^†a^
CCl_4_ + silymarin (100 mg/kg)	2.81 ± 0.10^a^	17.87 ± 1.01^≠bcd^	4.20 ± 0.27^†bc^	2.93 ± 0.20^*bc^	2.94 ± 0.21^cd^
CCl_4_ + FXM (200 mg/kg)	1.87 ± 0.13^†b^	14.62 ± 1.56d^†e^	4.65 ± 0.47^†b^	3.04 ± 0.25^≠b^	4.50 ± 0.34^†b^
CCl_4_ + FXM (400 mg/kg)	2.91 ± 0.11^a^	17.73 ± 1.00^≠cd^	3.80 ± 0.34^≠cd^	2.74 ± 0.35^bc^	3.38 ± 0.23^†c^
FXM (200 mg/kg)	2.90 ± 0.13^a^	21.74 ± 1.82^a^	2.95 ± 0.19^d^	2.51 ± 0.21^c^	2.55 ± 0.09^d^
FXM (400 mg/kg)	2.89 ± 0.11^a^	22.35 ± 1.65^a^	3.13 ± 0.33^d^	2.49 ± 0.18^c^	2.49 ± 0.16^d^

*FXM F. xanthoxyloides* methanol extract. Data values shown represent mean ± SD (*n* = 6). One way analysis of variance was followed by Dunnet comparison of various treatments with control at: ^*^, *P* < 0.05; ^#^, *P* < 0.01; ^ǂ^, *P* < 0.001. Multiple comparisons among treatments were determined by Tukeys’ HSD test. Superscript alphabets indicate significance among treatments (*P* < 0.01) on not sharing common letter

### Effect of FXM on comet parameters

**Fig. 2:**
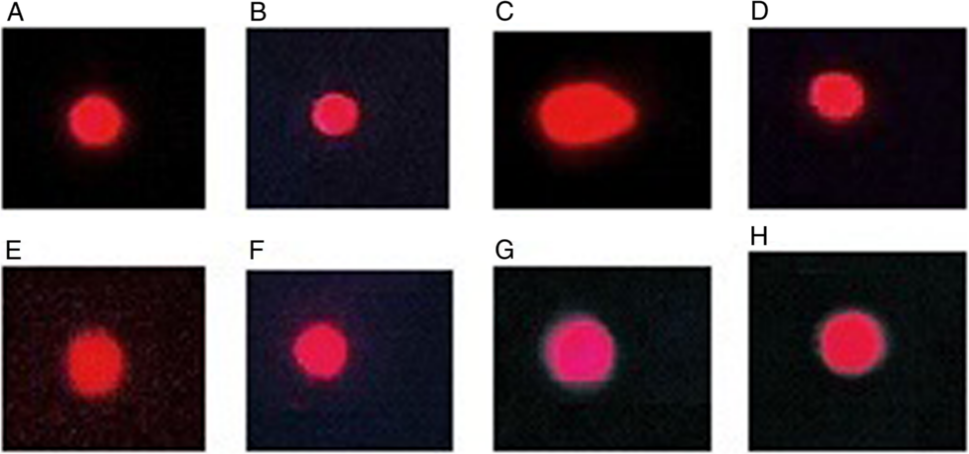
Comet studies of hepatic tissues. Ethidium bromide stain; 40× (**a**) Untreated control (**b**) vehicle treated (DMSO + olive oil) (**c**) CCl_4_ treated (**d**) CCl_4_ + silymarin (100 mg/kg) treated (**e**) CCl_4_ + FXM (200 mg/kg) treated (**f**) CCl_4_ + FXM (400 mg/kg) treated (**g**) FXM (200 mg/kg) treated (**h**) FXM (400 mg/kg) treated rats. FXM; *F. xanthoxyloides* leaves methanol extract

Table 4 shows the protective effects of FXM on the comet parameters after toxicity induced with CCl_4_ in hepatic cells of rat. The results obtained in this study indicated that the treatment of CCl_4_ induced the DNA injuries in hepatic cells and the comet length, head length, tail length, %DNA in tail and the tail moment was significantly (*P* < 0.001) increased as compared to the control group. However, %DNA in head was significantly (*P* < 0.001) decreased as against the control group. Prophylactic treatment of FXM alleviated the toxic effects of CCl_4_ and reversed the above parameters towards the control group. Multiple comparison among various treatments indicated that higher dose of FXM (400 mg/kg) significantly (*P* < 0.01) restored all the parameters of comet and its protective effects were comparable to the silymarin treatment. Administration of FXM alone did not alter (*P* > 0.05) the level of comet parameters as compared to the control group (Fig. 2).

**Table 4:** Effect of FXM on comet parameters of liver cells in rat

Treatment	Comet length (μm)	Head length (μm)	Tail length (μm)	DNA head (%)	DNA tail (%)	Tail moment (μm)
Control	33.26 ± 1.17^d^	26.55 ± 1.14^c^	6.71 ± 1.85^d^	96.81 ± 0.94^a^	3.18 ± 0.94^d^	0.51 ± 0.02^c^
DMSO + Olive oil	33.16 ± 1.03^d^	26.27 ± 0.36^c^	6.88 ± 1.29^d^	96.89 ± 1.00^a^	3.10 ± 1.00^d^	0.52 ± 0.01^c^
CCl_4_	65.21 ± 1.74^ǂa^	42.01 ± 2.02^ǂa^	23.20 ± 2.56^ǂa^	70.00 ± 1.54^ǂd^	29.99 ± 1.54^ǂa^	1.80 ± 0.10^ǂa^
CCl_4_ + silymarin (100 mg/kg)	40.79 ± 1.67^ǂc^	28.71 ± 1.01^*c^	12.07 ± 1.56^ǂbc^	89.81 ± 1.13^ǂb^	10.18 ± 1.13^ǂc^	0.60 ± 0.03^≠bc^
CCl_4_ + FXM (200 mg/kg)	49.25 ± 2.32^ǂb^	33.29 ± 1.19^ǂb^	15.96 ± 3.25^ǂb^	85.86 ± 1.68^ǂc^	14.14 ± 1.68^ǂb^	0.68 ± 0.02^ǂb^
CCl_4_ + FXM (400 mg/kg)	40.98 ± 1.68^ǂc^	26.86 ± 1.08^c^	14.11 ± 1.56^ǂb^	90.65 ± 1.32^ǂb^	9.35 ± 1.32^ǂc^	0.60 ± 0.02^≠bc^
FXM (200 mg/kg)	33.93 ± 1.60^d^	26.22 ± 0.99^c^	7.71 ± 2.44^cd^	94.74 ± 2.23^a^	5.25 ± 2.23^d^	0.53 ± 0.01^c^
FXM (400 mg/kg)	34.32 ± 1.33^d^	26.43 ± 1.41^c^	7.88 ± 1.60^cd^	95.37 ± 1.03^a^	4.62 ± 1.03^d^	0.53 ± 0.01^c^

*FXM F. xanthoxyloides* methanol extract. Data values shown represent mean ± SD (*n* = 6). One way analysis of variance was followed by Dunnet comparison of various treatments with control at: ^*^, *P* < 0.05; ^#^, *P* < 0.01; ^ǂ^, *P* < 0.001. Multiple comparisons among treatments were determined by Tukeys’ HSD test. Superscript alphabets indicate significance among treatments (*P* < 0.01) on not sharing common letter

### Effect of FXM on histopathology of liver

**Fig. 3:**
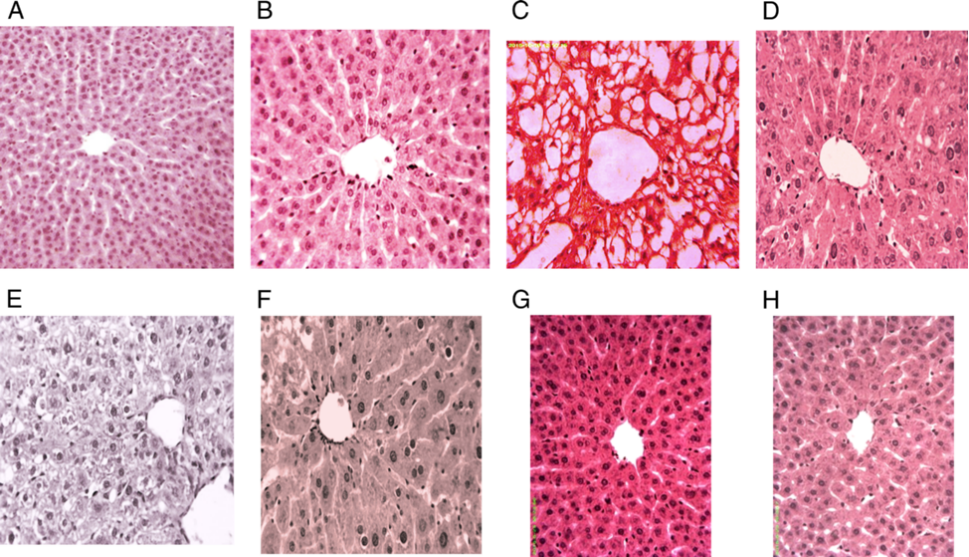
Histopathological studies of liver. Hematoxylin and eosin stain; 40× (**a**) Untreated control showing normal histoarchitecture of the hepatic tissues (**b**) vehicle treated (DMSO + olive oil) showing normal histoarchitecture of the hepatic tissues (**c**) CCl_4_ treated showing macrosteatosis (**d**) CCl_4_ + silymarin (100 mg/kg) treated showing almost normal histoarchitecture (**e**) CCl_4_ + FXM (200 mg/kg) treated showing microsteatosis (**f**) CCl_4_ + FXM (400 mg/kg) treated showing mild microsteatosis (**g**) FXM (200 mg/kg) treated showing normal histoarchitecture of the hepatic tissues (**h**) FXM (400 mg/kg) treated showing normal histoarchitecture of the hepatic tissues of rat. FXM; *F. xanthoxyloides* leaves methanol extract

Histopathology of the hematoxylin and eosin stained sections of hepatic tissues is presented in Fig. 3. Typical normal histology of liver parenchyma cells, sinusoids and central vein in untreated control and vehicle treated groups (Fig. 3a, b) was observed. Administration of CCl_4_ for 30 days caused severe liver injuries, marked increased in fatty changes, cellular hypertrophy, and necrotic foci, degeneration of the lobular architecture, macrosteatosis and congested blood vessels with disturbed epithelium (Fig. 3c). Co-administration of silymarin ameliorated the hepatic injuries and decreased the collagen deposition with very less or no fatty changes and maintained normal lobular architecture near to control group (Fig. 3d). Treatment of 200 mg/kg of FXM along with CCl_4_ represented hepatic injuries (Fig. 3e). However, the higher dose of FXM exhibited the hepato-protective potential near to control group (Fig. 3f). The histological observations are supporting the serological findings as well as biochemical studies in respect of oxidative stress. The administration of FXM alone did not induce histopathological alteration in liver tissues (Fig. 3g, h).

## Discussion

Foods rich in polyphenolics especially flavonoids are considered important nutraceuticals having health promoting effects in human. As flavonoids impart many medicinal properties, but perhaps the most important are the scavenging and chelating effects on free radicals. The HPLC analysis of FXM has indicated the presence of two flavonoids; rutin and caffeic acid. Rutin (quercetin-3-rhamnosyl glucoside), a natural flavone derivative exhibited several biological activities; antioxidant, antimicrobial, antiallergic, anti-inflammatory, vasoactive, antitumor [46–48]. Rutin has convincingly ameliorated the toxic effects of CCl_4_ and has shown the hepato and nephroprotective abilities [49, 50]. It was investigated that the expression of CYP2E1 in liver cells of rat was decreased by the administration of CCl_4_ to rats. However, the level of CYP2E1 was not affected by the treatment of CCl_4_ metabolites such as trichloromethyl and trichloromethyl peroxy radicals. The decrease in the expression level of CYP2E1 might occur through the production of active metabolites of CCl_4_ by an independent CYP2E1 pathway [51]. The downregulation of CYP2E1 might account the over expression of inflammatory mediators. The decrease in CYP2E1 expression induced with CCl_4_ in liver of rat was restored with the administration of rutin [50]. Caffeic acid reduces the acute immune and inflammatory response [51]. Caffeic acid also possesses antitumor abilities [52]. It has also been illustrated that these compounds are strong antioxidants, in which caffeic acid is considered to be the highest antioxidant due to the dihydroxylation of 3, 4- position on the phenolic ring of caffeic acid [53]. Further, the HPLC-DAD analysis revealed the existence of other phenolic compounds as well (Fig. 1). FXM and its derived fractions exhibited anti-inflammatory activities both *in vitro* and *in vivo* studies by lowering the concentration of inflammatory mediators including nitric oxide. GC-MS analysis of FXM indicated the presence of squalene, 2-linoleoyl glycerol, 2-palmitoyl glycerol anti-inflammatory compounds which prevent activation of macrophages, neutrophils and monocytes [35]. Liver is directly affected by the release of CCl_4_ metabolites and cytokines that propagate inflammatory response [54]. Antioxidant capacity of FXM in this study might be attributed by the existence of rutin, caffeic acid and other antioxidant and/or anti-inflammatory compounds in protecting the biological system against the potentially harmful effects of free radicals.

Three pure compounds; rutin and nummularic acid from ethyl acetate fraction and plectranthoic acid from chloroform fraction of FXM were isolated and characterized by NMR and other spectroscopic analyses (data not shown). Nummularic acid and plectranthoic acid are the triterpenoids, a class of phyto-chemicals. Use of plectranthoic acid during *in vitro* studies has 5'AMP-activated kinase (AMPK) activity that is considered to be a metabolic hub for the treatment of type-2 diabetes and cancer [55]. Further GC-MS analysis of the FXM indicated the presence of various compounds belonging to 15 major classes of which; three were terpenoids (26.61 %), four lactam (16.47 %), three esters (15.81 %), three phenols (8.37 %), two steroid (6.91 %), three alcohols (5.02 %), three ketones (4.49 %), one aldehyde (3.89 %), two fatty acid glycerol (3.01 %), one nitrile (2.64 %), two lactones (2.31 %), one silyl-ether (2.25 %), one alkene (1.31 %) and one alkyne (0.89 %) [35].

Jaundice is also included among the inflammatory diseases induced with free radicals. ROS are recognized as the main factors involved in DNA damages, lipid peroxidation and protein injuries. Liver is highly vulnerable to chemical toxicity and liver cirrhosis as one of the dominant processes that lead towards the death of the organism. A number of studies have indicated that liver damages can be induced in experimental organisms by administration of CCl_4_. Metabolites of CCl_4_ namely trichloromethyl and trichloromethyl peroxy radicals are produced during its metabolism by cytochrome P-450, and imposes oxidative stress that cause widespread liver damages. Leaves of *F. xanthoxyloides* are used locally in the treatment of various disorders including jaundice [15].

Recent studies have indicated the potential therapeutic importance of plant derived products against the oxidative stress induced hepatic anomalies [12, 56]. Estimation of the liver function enzymes; AST and ALT, total bilirubin, albumin and globulin represent the quantitative marker of the hepatocellular damage and the metabolic integrity of the liver. In this experiment the increased level of AST, ALT, total bilirubin and globulin while deceased level of albumin in serum indicated the severe hepatocellular damages induced with CCl_4_ intoxication. The enzymes; AST and ALT are cytoplasmic in location and the significant increase in the level of AST and ALT in serum of CCl_4_ treated group might occur as leakage or necrosis of the hepatic cells reflecting lipid peroxidation of cellular membranes. Severe hepatic damages induced with CCl_4_ intoxication depressed the function of liver. Significant increase in globulin while the decrease in albumin indicated the extensive damages induced with CCl_4_ that led to the reduced synthesis of albumin. Decrease in albumin can also be accounted due to enhanced proteinuria as reported in various studies [57, 58]. However, prophylactic treatment of FXM restored the liver function enzymes, total bilirubin, albumin and globulin in serum. These results suggested the therapeutic role of FXM against the oxidative damages. The restoration of the above parameters might occur due to prevention of oxidative injuries and stabilization of the cellular membranes [59].

In this experiment activity level of antioxidant enzymes; CAT, POD, SOD, GST and GSR of hepatic tissues was significantly decreased indicating that the oxidative stress was induced with the treatment of CCl_4_ in rat. It is an accepted view that the coordinated action of antioxidant enzymes is a prerequisite for scavenging of deleterious free radicals. The overwhelming and perpetual generation of free radicals suppresses the activities of antioxidant enzymes. The superoxide radicals generated during the normal metabolism and/or during intoxication are converted by the SOD enzyme in to H_2_O_2_ which is subsequently decomposed by CAT and glutathione peroxidase thus eliminating the potential hazardous effect of hydroxyl radicals to the tissues. In this investigation the treatment of CCl_4_ to rats elevated the concentration of H_2_O_2_ in hepatic tissues. The severe hepatic injuries observed in this experiment with CCl_4_ provide a link that OH radicals generated during the conversion of H_2_O_2_ can abstract hydrogen from the poly unsaturated fatty acid of the membranous system. Further, the compromised activity of antioxidant enzymes deteriorates the situation by generation of secondary free radicals. The results presented in this study are instigated with other studies [59, 60] where the CCl_4_ intoxication has increased the level of H_2_O_2_ and suppressed the activity of antioxidant enzymes in hepatic tissues. The rats exposed to CCl_4_ and with FXM simultaneously exhibited significant increase in the activity level of antioxidant enzymes along with significant decrease in H_2_O_2_ of sample homogenates as compared to the CCl_4_ group. These results support the proposed mechanism that CCl_4_ induces oxidative stress in hepatic tissues by generation of free radicals whereas the co-administration of FXM alleviates the oxidative stress by scavenging of free radicals and led to the restoration of antioxidant enzymes. These resulted have been supported by other studies where the co-administration of antioxidant agents have ameliorated the toxic effects of CCl_4_ and brought back the activity level of antioxidant enzymes [60].

In the present studies the toxicity of CCl_4_ led to a significant increase in lipid peroxides (TBARS) while significant decrease in GSH in liver homogenate samples as compared to the control group. Treatment of CCl_4_ induces oxidative stress in hepatic tissues by generation of free radicals and is implicated in lipid peroxidation [60]. Estimation of lipid peroxides in the sample is an effective approach to verify the presence of hepatic anomalies. Restoration of TBARS in hepatic samples by co-administration of FXM in this study indicated the protective potential against the CCl_4_ induced hepatic injuries in rat. These results have been verified by obtaining lower damages during histopathological studies. GSH plays a crucial role during scavenging of free radicals especially during the decomposition of H_2_O_2_ and scavenging of hydroxyl radicals. CCl_4_ provoked free radicals are detoxified by forming conjugates with GSH and this property of GSH render it to play a major role in elimination of CCl_4_ induced toxic metabolites. The availability of GSH dispensed the maintenance of oxidants and its depletion may result in severe hepatic injuries. In this study treatment of FXM restores the concentration of GSH that provides a plausible mechanism of hepato-protection against oxidative injuries [59, 60].

Lipid peroxidation is a dual process not only involved in the destruction of membranous system of the cell but is also involved in the synthesis of highly reactive hydro peroxides having enormous ability to interact with proteins and even DNA. The enhanced level of DNA injuries in hepatic tissues was recorded with the treatment of CCl_4_ to rats. The results obtained in this study are in line to a previous study where the DNA fragmentation and the major oxidative lesion 8-oxo-2-deoxyguanosine (oxo8dG) were increased in liver with CCl_4_ treatment and reverted towards the control level with co-administration of rutin to rats [49]. Oxo8dG in DNA induces misreading during DNA synthesis *in vitro* and leads to G → T transversion mutagenesis. Studies of comet assays of the hepatic cells have endorsed the DNA injuries induced with CCl_4_ metabolites. There has been an increase in the comet length, head and tail length and %DNA in tail in cells of liver samples. The increase in these parameters might reflect the DNA breaks and the damages of the membranous system of the hepatic cells. Such injuries might augment the hepatic injuries and functional anomalies. The results recorded in this study were in conformity to Sajid et al. [61] where the comet length had increased while the %DNA in head was decreased with CCl_4_ treatment to rats. However a restoration in these comet parameters was recorded with co-administration of methanol extract of *Artemisia scoparia* [61].

## Conclusion

The altered biological profile of hepatic tissues induced with CCl_4_ was restored towards the control level by the co-administration of FXM and silymarin, a standard antioxidant drug to rats. These results suggested the repairing as well as regenerative capacity of the antioxidants present in FXM.

## Abbreviations

ALT: Alanine transaminase
AST: Aspartate transaminase
CAT: Catalase
CCl_4_: Carbon tetrachloride
FXM: *Fraxinus xanthoxyloides* methanol extract of leaves
GSH: Rerduced glutathione
GSR: Glutathione reductase
GST: Glutathione-S-transferase
POD: Peroxidase
SOD: Superoxide dismutase
TBARS: Thiobarbituric acid reactive substances
